# More, better feedback please: are learning analytics dashboards (LAD) the solution to a wicked problem?

**DOI:** 10.1007/s10459-024-10358-8

**Published:** 2024-08-26

**Authors:** Simon Kitto, H. L. Michelle Chiang, Olivia Ng, Jennifer Cleland

**Affiliations:** 1 Lee Kong Chian School of Medicine, Nanyang Technological University, HQ Building, Novena Campus, 11 Mandalay Road, Singapore, 308232 Singapore; 2 National Healthcare Group (NHG), Singapore, Singapore

**Keywords:** Educational technology, Feedback, Learning analytics, Digital dashboards, Health professions education

## Abstract

There is a long-standing lack of learner satisfaction with quality and quantity of feedback in health professions education (HPE) and training. To address this, university and training programmes are increasingly using technological advancements and data analytic tools to provide feedback. One such educational technology is the Learning Analytic Dashboard (LAD), which holds the promise of a comprehensive view of student performance via partial or fully automated feedback delivered to learners in real time. The possibility of displaying performance data visually, on a single platform, so users can access and process feedback efficiently and constantly, and use this to improve their performance, is very attractive to users, educators and institutions. However, the mainstream literature tends to take an atheoretical and instrumentalist view of LADs, a view that uncritically celebrates the promise of LAD’s capacity to provide a ‘technical fix’ to the ‘wicked problem’ of feedback in health professions education. This paper seeks to recast the discussion of LADs as something other than a benign material technology using the lenses of Miller and Rose’s technologies of government and Barry’s theory of Technological Societies, where such technical devices are also inherently agentic and political. An examination of the purpose, design and deployment of LADs from these theoretical perspectives can reveal how these educational devices shape and govern the HPE learner body in different ways, which in turn, may produce a myriad of unintended– and ironic– effects on the feedback process. In this Reflections article we wish to encourage health professions education scholars to examine the practices and consequences thereof of the ever-expanding use of LADs more deeply and with a sense of urgency.

## Introduction

Effective feedback has long been recognised as a fundamental catalyst for effective learning (Butler & Winne, 1995; Hattie & Timperley, 2007). However, learners in higher and health professions education (HPE) are consistently dissatisfied with feedback and report feedback provision as insufficient, a notion that is consistently disputed by supervisors and that many interventions to improve feedback delivery have not been wholly successful in rectifying (Boud & Molloy, 2013; Carless, 2006; Deeley et al., 2019; Ossenberg et al., 2019).

Progress in educational technologies and learning data analytics offer new opportunities to structure and deliver personalised feedback efficiently to learners. Indeed, over the last few decades, the higher education literature has been characterised by critical commentary and research on how educational technologies have the potential to, and are already, reconfiguring relationships between lecturers and students, and altering the learning behaviour of student learning itself (Kitto, 2003). In higher and health professions education, the potential of educational technologies such as learning analytics, machine learning and artificial intelligence (AI) is treated with optimistic caution (Kitto et al., 2024). But what is largely absent from this literature is a theorization of the nature of such technological tools in terms of their larger role in the organization of social order, and more specifically, in the conduct of health professions education itself.

Our focus in is on one specific learning technology, that of student-facing, technology-mediated, learning analytics dashboards (LADs). As a Reflections contribution, this paper does not adhere to the traditional scientific format of introduction, methods, results and discussion. Rather it represents a summary of many discussions held over time, with reference to a wide literature and the many considerations we have grappled with as part of developing and implementing a LAD locally.

LADs are positioned within the literature as a feedback intervention (Clow, 2013), usually presented as: “single displays that aggregated different indicators about learners, learning processes and or learning contexts into one or multiple visualizations”, so the information can be monitored at a glance (Schwendimann et al., 2017). At its most basic, a LAD employs descriptive analytics to provide an overview of a learner’s progress whereas a state-of-the-art LAD (at the time of writing this paper) integrates multiple data sources (e.g., assessment, attendance, clinical skills checklists data, etc.) and multiple analytical levels (e.g., (Few, 2007); see also (Boscardin et al., 2018), and discussed further later).

**Fig. 1:**
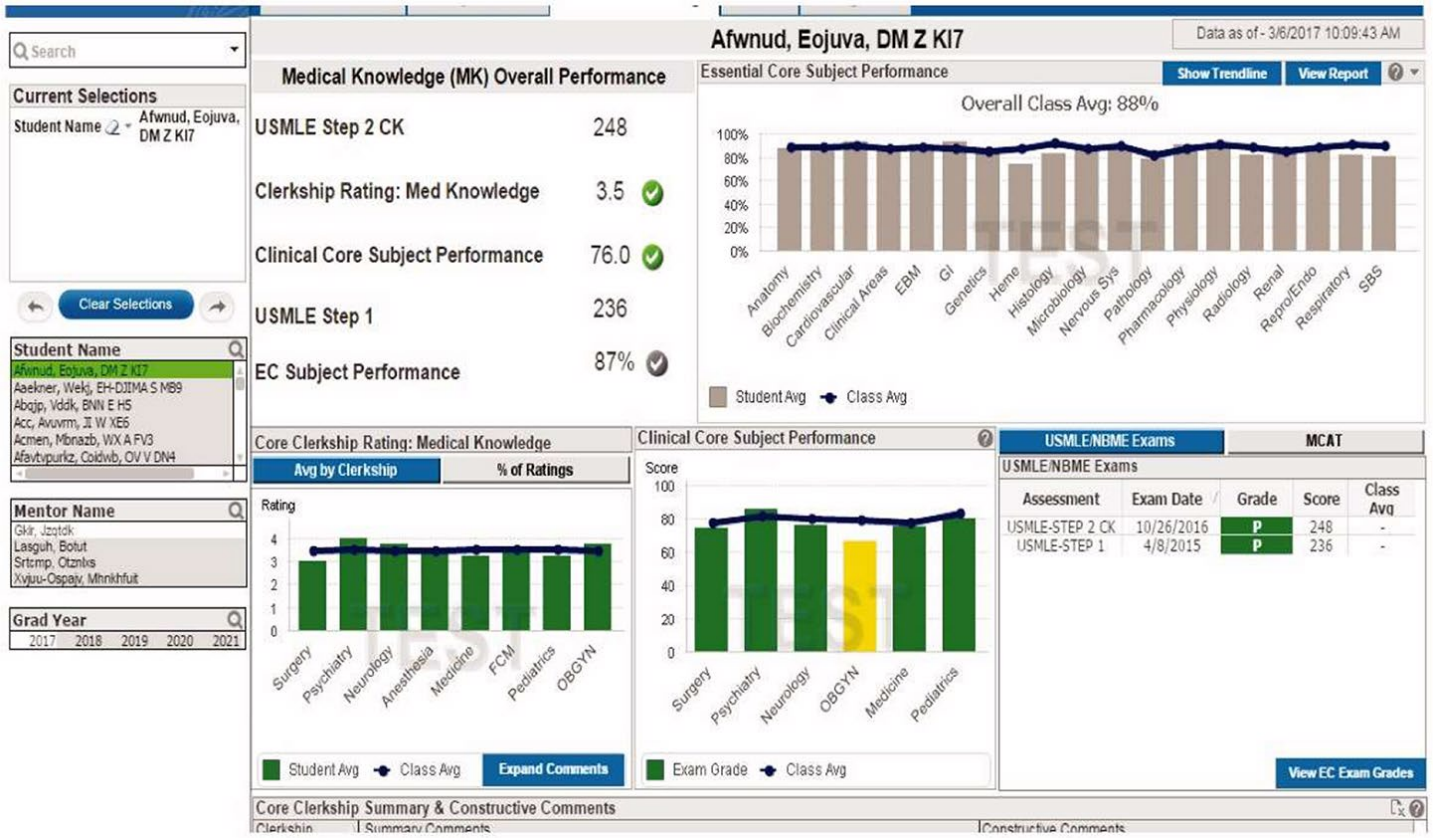
An example Learning analytics dashboard (LAD) in medical education that presents a comprehensive, visual overview of student progress (Boscardin et al., 2018)

To illustrate what we mean by a LAD, we have drawn upon a combination of figures and descriptions found in the literature and ‘case study’ examples we created from an amalgam of possible LAD design possibilities (see Fig. 1 for an example of a LAD).

For learners, LADs are posited to facilitate self-reflective learning through visualization, highlighting key performance moments and often comparing individual performance to class averages (Susnjak et al., 2022). For instructors, LADs construct insights into learners’ performance and progress, aiding academic advising and study plan development (Gutiérrez et al., 2020). Some LADs also employ predictive, machine learning algorithms to detect potentially problematic behaviours indicative of being at-risk and generate academic recommendations, such as remediation suggestions based on identified academic issues. At the institutional level, data gathered from a LAD could aid in the customisation of educational strategies that would meet regulatory or accreditation needs. In short, by integrating multiple data sources and analytical levels about student performance LADs are meant to provide a deeper understanding and better facilitation of students’ learning processes, enhance communication, support decision-making, and improve academic outcomes for both learners and instructors (Bodily & Verbert, 2017; Masiello et al., 2024). In short, LADs provide, or are purported to provide, high quality feedback.

In general, the LAD literature has been quite limited, focusing on dashboard architecture and components (Bodily et al., 2018), design and technical considerations, and the lessons learned in implementation (Durojaiye et al., 2018; Herodotou et al., 2019); the needs and ways of linking dashboard content and visualization and learning science concepts to improve the accuracy and effectiveness of LAD (Sedrakyan et al., 2019; Teasley, 2018); and, in health professions education (HPE), the development and implementation of a LAD (Boscardin et al., 2018). While there are some recent exceptions in the wider literature (e.g., (Paulsen & Lindsay, 2024), taken as a whole, this body of research to date tends to frame the promise of LADs in an instrumental mechanistic manner, as an efficient and rather benign technological approach to feedback (Banihashem et al., 2022). There is little to no critical analyses of what role and effects a LAD might have on learner performance and learner relationships with their peers, instructors or the educational institutions in which they are enrolled. Rather the literature has a seductive tone, suggesting LADs can provide all the promises of a ‘technical fix’ (Robins & Webster, 1989) underpinned by a belief that ‘wicked’ (Rittel, 1973) educational problems like feedback (Deeley et al., 2019) can be resolved through the application of a technological artifact (that is, a LAD) without any unintended consequences.

We attempt to address this gap in the literature using the lens of the technological society (Barry, 2001). Using Barry’s heuristic framework as a conceptually coherent means by which to explore the relationships between LADs and the governing of medical education, we open discussion as to the role and effects LADs might have, how they might act directly on the student and instructor(s).

First, we outline the concept of the Technological Society and link this to the explicit agenda of encouraging self-governance of medical students through information technology, in this case LADs. We will show how using this conceptual framework can serve to unpack the potential impact of the assumptions behind, design of the functions and deployment of LADs on medical student populations. Specifically, we will consider how a LAD, as a technical device, acts as a political technology (see later) in the shaping of the learning relationship between technology, student and lecturer, and the downstream effects this reshaped relationship may have on student behaviour. Before doing so, we reflect on our interest in this topic.

### Our positionality

Positionality “reflects the position that the researcher has chosen to adopt within a given research study” (Savin-Baden & Major, 2013, p.71). It influences both how research is conducted, its outcomes, and results (Rowe, 2014). The study was initially developed from ongoing, local discussions about introducing programmatic assessment (PA) (Schuwirth & Van der Vleuten, 2011) into a medical undergraduate degree programme (an MBBS) and presenting the assessment information collected from various sources to learners via a LAD. As part of this process, we accessed a wide range of literature about LADs, and were surprised at its lack of criticality and theorization.

We considered our positions and relationships with this literature and our early stage “on-the-ground” experiences of developing a LAD for local purposes (which at the time of the drafting of this article was yet to be completed and evaluated) continually and critically in view of: our disciplinary backgrounds (psychology but working in medical education [JC], sociology [SK], engineering [ON], English [MC]), levels of knowledge and perspectives on assessment and feedback in health professions education and learning analytics. For example, a large part of ON’s role was to support the development and implementation of LADs locally, so she brought much understanding of the possibilities and limitations of learning analytics and visualisation thereof. JC, MC and SK had less technical expert knowledge and more “etic”, critical views.

### Positioning LADs within a technological society

The relationship between information technologies and governing contemporary western societies has been widely explored. More than 30 years ago, inspired by the ideas and works of Michel Foucault on the operations of power in modern society, Peter Miller and Nikolas Rose (Miller & Rose, 1990; Rose & Miller, 1992) alluded to the infrastructural nature of information technologies through an exposition of the nature of political rationalities and technologies of government. Political rationalities are the discourses where power is exercised and where moral justifications are made for the exercise of that power (Rose & Miller, 1992). When a problematic aspect of governing is identified (e.g., student dissatisfaction with feedback quality and quantity), political rationalities are translated into technologies of government that shape sectors of society into desirable, implementable forms (Miller & Rose, 1990). Through these technologies, authority seeks to “shape, normalize and instrumentalize the conduct, thought, decisions and aspirations of others” (Miller & Rose, 1990, p.8) while reaching their own objectives and desires (e.g., designing the LAD in a certain way to direct how feedback is delivered, how learners interact with the dashboard in order to acquire knowledge and skills to become competent professionals in the workplace).

Andrew Barry’s concept of the Technological Society places information technology at the centre of governing, where citizens must now actively use and interact with technology to maximise their choices and demonstrate mastery and self-responsibility over the conduct of their lives (Barry, 2001). Using this lens, a LAD is conceptualised as being inherently political, and functions as a technology of governing in the Foucauldian sense of creating the conditions of possibility in which individuals can conduct themselves (Foucault, 1991; Hamann, 2009). Positioning an LAD in a technology society highlights its dual nature, it is both:

‘…a technical device, conceived of as a material or immaterial artefact, and a technology, a concept which refers not just to a device in isolation but also to the forms of knowledge, skill, diagrams, charts, calculations and energy which makes its use possible’ (Barry, 2001, p.9) (see also (Akrich, 1992; Deleuze, 1988)).

This notion of technical devices as instrumental material objects which have technological capacities (governmental) and their mutability when translated into action, has been utilised to study socio-technical configurations of educational delivery, use by faculty, and reception and use by students within the higher education sector. These studies have found unintended effects of technological devices ranging from: purportedly unethical behaviour of students in the learning and assessment process, re-organisation of the relationship between student, faculty and the university, ironic performances of student freedom in association with educational technologies, and at times adverse re-representation of student behaviour and concomitantly the construction of student subjectivity (i.e., ‘good’ or ‘bad’) (Kitto, 2003; Kitto & Higgins, 2003, 2009, 2010; Kitto & Saltmarsh, 2007). For example, think of the advent of online education within the higher education sector in the late 1990s. Designed to democratise education by overcoming the tyranny of distance and to facilitate lifelong learning amongst adult learners, online education was position as a solution to many changes in society impacting the delivery of higher education: threats of globalization, the increase in use of information technologies to deliver education, emphasis on life-long learning, changes in the demographics, and needs/choices of students. Formerly disenfranchised learners (full-time workers, rural impoverished populations) were now being positioned to access top ranked tertiary education more cost effectively at a distance and in their own time frames (Robins & Webster, 2002). However, while such technical devices can enact political programs at a distance, they can also act produce outcomes that act against their intended political objectives (Barry, 2001). In the online education sector this took the form of the rise of disreputable ‘digital diploma mills’ (Noble, 1998), leading to reputational damage which still plagues the sector on a global scale.

### Learning analytics dashboards in health professions education

LADs supposedly offer a holistic view of a learner’s progress, consolidating visualised assessment data points on a one-stop information learners’ hub (Boscardin et al., 2018). The claim is that a LAD facilitates learners and educators to better understand individual learner performance by incorporating assessment (summative and formative), attendance, clinical skills checklist data, and so on. Having the ability to visualise connections between competencies and assessment ostensibly also allows for customisation of educational strategies based on accreditation needs (Chan et al., 2018). This capacity is made possible through the different levels of learning analytics include descriptive, diagnostic, predictive, and prescriptive analytics, each offering unique insights into data patterns and facilitating informed decision-making. We discuss these different levels of learning analytics in turn.

### Diagnostic and descriptive analytics: a panoptic technique within a technological society

The most common, and simplest function and form of feedback is descriptive analytics. For example, (Han et al., 2021) developed a student dashboard that informed students about their engagement levels and interactions with peers. Diagnostic analytics supposedly adds an extra dimension to descriptive analytics by offering insights into why something occurred, typically by discerning patterns and trends within the data. For example, (Aljohani et al., 2019) presented an LAD that utilized log data from a Learning Management System (LMS) to uncover student behavioural patterns and attitudes. It compared each student’s engagement level with that of their peers, offering personalized learning statistics along with comparisons to class averages and top-performing peers, to promote self-awareness and aid in performance assessment.

**Fig. 2:**
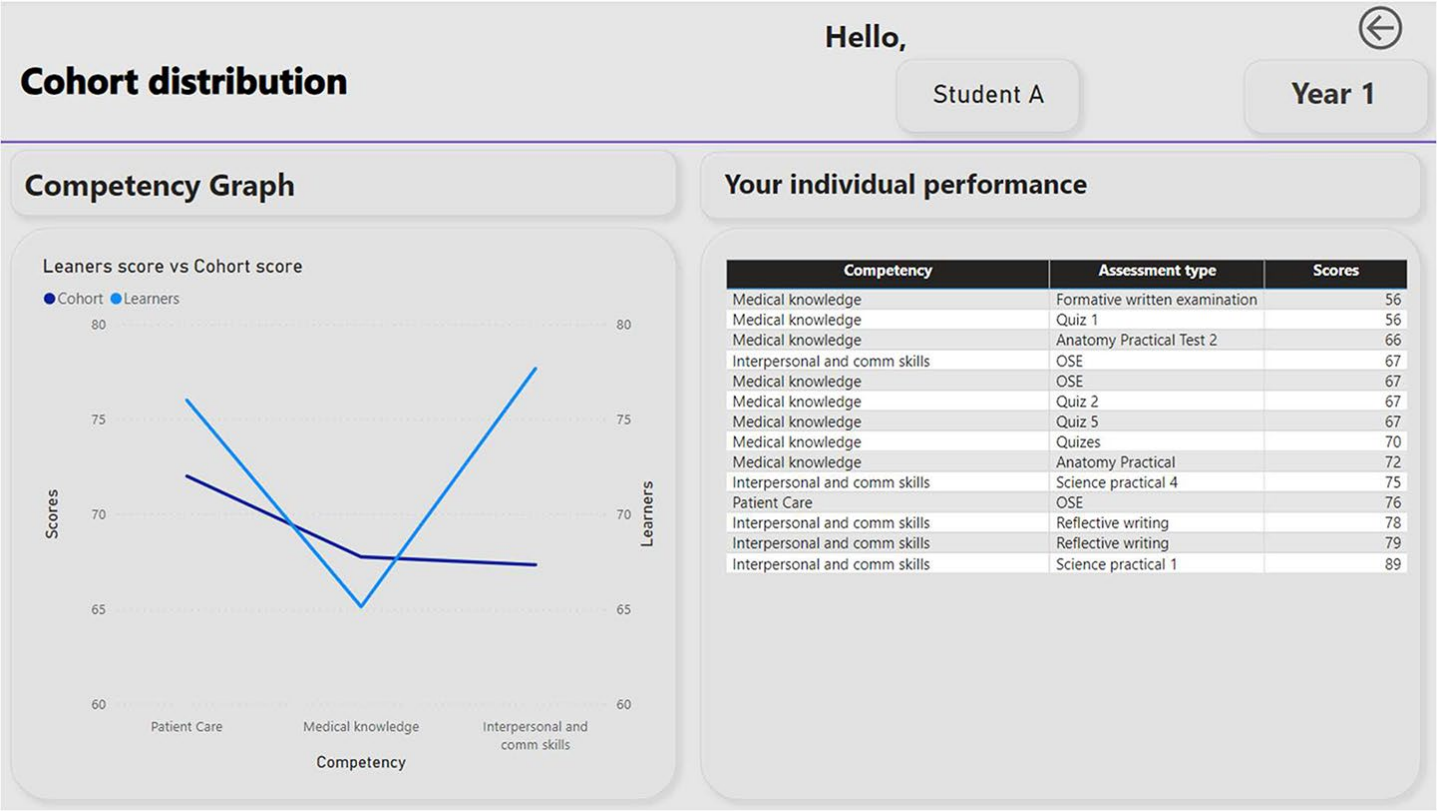
Individual - Cohort comparison presented in a hypothetical LAD

Applying a technological society lens to these functions highlights the manifestation of three political functions and techniques of government: (1) in a technological society the notion that students, via technological means, are expected to *interact* in a self-governing way is a core political rationale, (2) through this interaction they are expected to come to know themselves, through technological intermediaries, in order to be able to work on themselves, (3) to do this they must direct that interaction by placing themselves in an extended web of connections with other people, institutions and forms of knowledge (Barry, 2001). In this case, the LAD already places them within such a web of connections through the combining of diagnostic and descriptive functions. Supposedly high-fidelity data about a student’s performance is analysed, compiled, (re)represented in visual form to show individual performance and how their progression sits in relation to a class of students (see Fig. 2).

In this respect, a LAD contains all the hallmarks of a Foucauldian panopticon, an ‘electronic panopticon’ (Kitto, 2003):

It refers individual actions to a whole that is at once a field of comparison, a space of differentiation and the principle of a rule to be followed. It differentiates individuals from one another…. It measures in quantitative terms and hierarchises in terms of value the abilities, the level, the ‘nature’ of individuals. It introduces, through this ‘value-giving’ measure, the constraint of a conformity that must be achieved.. it compares, differentiates, hierarchises, homogenizes, excludes. In short, it normalizes. (Foucault, 1977, p.182–183).

A LAD could be designed to simultaneously provide this type of a ‘normalising gaze’ to enable representatives of the medical school to work on student interventions when needed, and for the student, to give a picture of their ‘normality’ within their cohort (See Fig. 2). The LAD, or more accurately the data presented by the LAD, could act as the starting point for intervention by the school, by the student themselves in isolation, or in partnership with one another to improve the student performance.

**Fig. 3:**
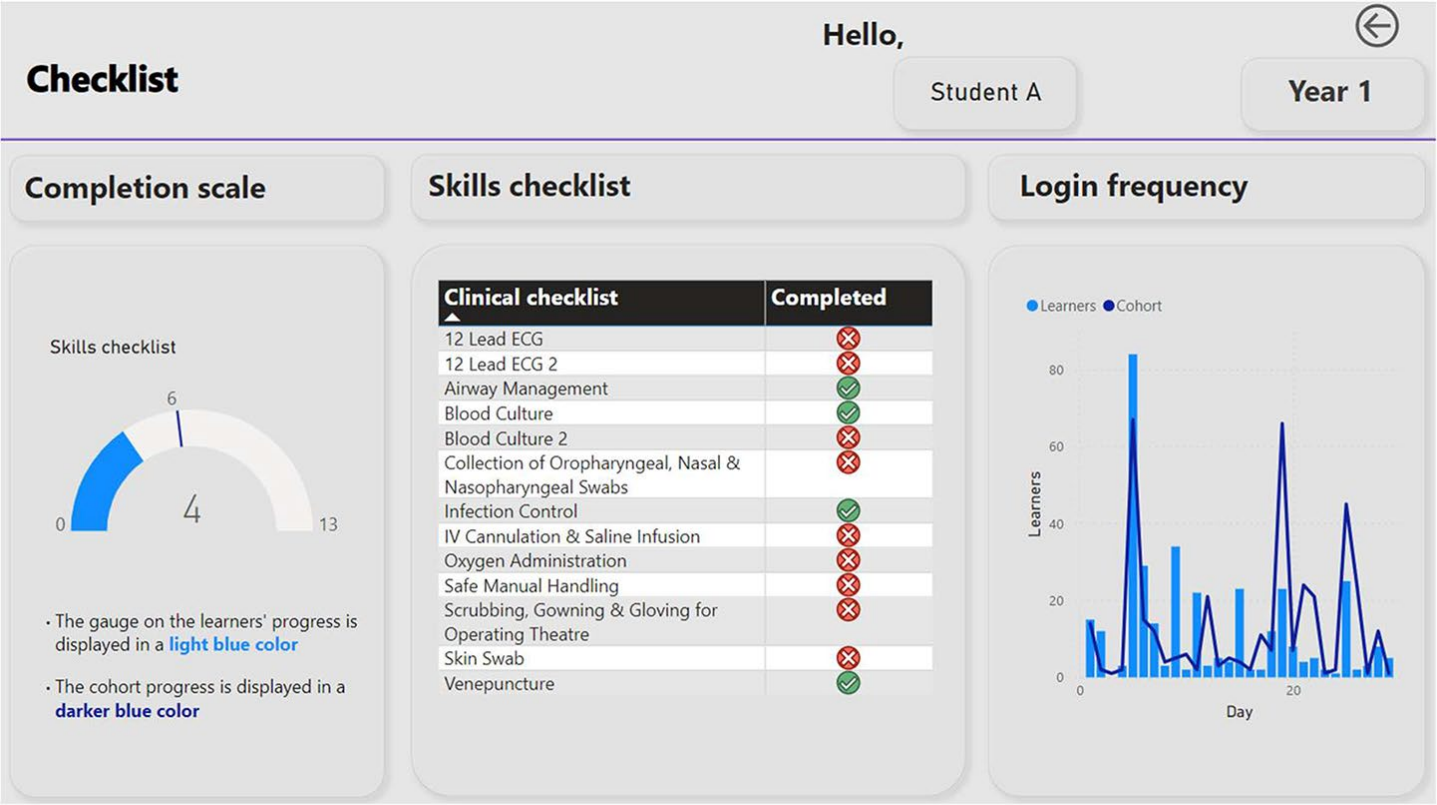
Student log data and completion rates visualised via a hypothetical LAD

This notion of the construction of panoptic techniques in education via technical means to normalise student populations is a well-worn scholarly path (Foucault, 1977; Kitto, 2003). What is new here is when seen through a Technological society lens, the problematic aspect of the combination of panoptic techniques under the conditions of governing via information communication technologies in a Technological Society becomes apparent. Now, multiple forms of data are connected to provide a ‘diagnosis’ not only of performance in student examinations, but of levels of *interactivity* within their curricular activities and with the LAD itself. Are the students completing all their activities? Are they *interacting* with enough frequency with the LAD (made visible by student log data) - as a demonstration of being a good interactive technological student-citizen seeking self-improvement? (see Fig. 3).

LADs are not simple techniques of a disciplinary society in the Foucauldian sense (you *must* do this). In a governmental technological society, they are advanced liberal technologies that seek to create self-governing capacities of students (you *may* do this) through regulated choices. The conditions of possibility for optimal self-governance (Rose, 1993), are provided by LADs by managing and moulding learners in specific ways. How the LADs data is used by those who govern is, of course, dependent on the local cultural and structural idiosyncrasies of the medical school itself (predilections of leaders, accreditation pressures etc.). Think for instance, of remediation sessions recommended for ‘recalcitrant’ students for not completing the skills checklist. The threshold for recommending such, and precisely what is recommended, will differ from institution to institution: governing via technical means is always context bound (Barry, 2001).

### Predictive and prescriptive analytics in LADs

Two more functions - predictive and prescriptive analytics - provide a further layer of complexity, making LADs something more than a simple panoptic technique to shape student population and individual behaviour. They are also saturated with ways of thinking about how to provide the optimal conditions for the student-citizen to self-govern, which these student-citizens must learn to navigate to properly self-govern.

Under advanced liberal government, the ‘free’ citizen ironically, is always bound in time and space within assemblages of governing that set standards to evaluate their performance as well as that of institutions (think medical school accreditation standards and practices). This requires a system of navigation for the individual/institution to properly self-govern, it requires expertise and experts:

Certain, civilised modes of conducting one’s existence are identified as normal, and simultaneously to be bound to those ‘engineers of the human soul’ who will define the norm and tutor individuals as the ways of living that will accomplish normality. (Rose, 1999, p.76).

With predictive and prescriptive analytics, this relationship becomes quite complex. Predictive analytics uses current or past data to inform future outcomes– often through machine learning algorithms. Rather than simply presenting raw log data, many dashboards process this information using machine learning models or algorithms to provide insights (Afzaal et al., 2021; Gutiérrez et al., 2020). For example, Herodotou et al. (2019) used LMS log data to predict student assignment submission and course completion, and to identify those considered “at risk” (see also (Gutiérrez et al., 2020; Mavrikis et al., 2019)) (the last being a constant pre-occupation in advanced liberal societies).

Prescriptive analytics aims to provide solutions for determining what actions should be taken. They tend to utilise predictive analytics to infer potential actions leading to positive outcomes, often in the form of recommendations based on data collected from various sources such as LMS. Examples include real-time recommender-type dashboards introduced by (Bodily et al., 2018) and (Sansom et al., 2020).

As previously mentioned, a key aspect of advanced liberal ways of rule in technological societies is to enact programmes of government that can create the conditions of possibility for individual active and self-responsiblized comportment (Barry, 2001). What is interesting in the above description of the predictive and prescriptive functions of a LAD and its design as a technology of governing is that it seemingly sets up the foundation for a contradictory interplay between two key diagrams of social organisation, that of a panopticon (Foucault, 1977) and oligopticon (Latour, 2005). As previously outlined, the panopticon is a political technology that serves to individualize and normalize through a system of continual clear surveillance, characterized by classification and judgement. It governs populations by instilling compliance and optimize the capacities of the individual to maximize their social and economic utility (Rose, 1999). The panoramic view of the panopticon (and the knowledge of its existence by the observed) is in stark opposition to the oligopticon, which consists of multiple narrow views of a complex and connected landscape that can be made blind by the smallest disruption (Gad & Lauritsen, 2009). Inherently fragile, “they see much too little… but what they see, they see it well… sturdy but extremely narrow views of the (connected) whole are made possible—as long as connections hold (Latour, 2005, p.181). In an information technology context, the success of oligopotic ‘surveillance is the result of situated, cooperative work that involves humans and nonhumans. Effective surveillance is not established by an individual actor but is accompanied by a network’ (Gad & Lauritsen, 2009, p.53).

The fragility of these connections when the LAD is deployed as a mechanism of feedback is everpresent. Let’s look more closely at the rationale behind the communication channel or feedback mechanism of LADs. The aim is enhancing personalized learning through the full or partial automation and streamlining of feedback processes. Usually, the goal is to institute this longitudinally throughout a programme of learning, such as through a medical degree, with the potential for expansion to the residency level (postgraduate) and the selection process. The idea is that this would provide a representation of one’s medical education journey, aiding students in understanding themselves and their educational needs. This is characteristic of a technology of governing in a Technological society where the political rationality behind the key mechanism of organizing the activities of individual citizens, is self-directed lifelong learning that is supported by “constant forms of feedback” that are technologically mediated (Barry, 2001). But like all technologies of governing, it is a ‘congenitally failing operation’ (Miller & Rose, 1990) and thus, “different problems of context or locale or knowledge will have to be taken into account” (Barry, 2001, p.16).

A LAD can visualise connections between competencies and assessment (see Fig. 2). While a tantalising proposition for educators, this assumption fails to examine who or what is making the connections that form the visual displays from which students can become ‘experts’ of themselves. The power of the display is made manifest by algorithms interpreting the data and imputing meaning onto the connections which are, through interaction with dashboard, subsequently situated within the body of the student by the student themselves and at times, with assistance by faculty in the form of feedback. This is a complex socio-technical assemblage in that it contains relationships between bodies and machines that can act as multiple translation points in different moments within multiple forms of feedback performances, such as– (1) individual student interacting with the LAD to ‘assess’ their performance and interpret automated feedback (2) clinical instructor interacting with the LAD to ‘assess’ the student’s performance and engage in a further interpretation of the automated feedback (3) student and instructor interacting together with the LAD to construct a shared meaning of the visual displays of ‘assessment’ of performance and automated recommended learning needs.

In socio-material terms the performance of these socio-technical assemblages creates a representation of the student’s bodily performance, but as ‘social agents [students] are never located in bodies and bodies alone’ (Law, 1992, p.382–384), they are cyborgs, human and non-human hybrids (Harraway, 1991) that are stabilized and ‘purified’ (Latour, 1993) as human or technical in different points in time and space. Often they are ‘purified before [we] even have the chance to interrogate their hybridity’ (Michael, 1998, p.134) This directly effects how the translation and interpretation of human practices via databases which are abstractions from actual bodily practices (see (Lyon, 2001), are shaped and used in the process of governing populations and the training of individuals within Technological Societies (Barry, 2001; Deleuze, 1992; Poster, 1990). If the student’s performance is an outcome of an array of bodies, representational devices such as databases, textual and visual displays, alongside actual in situ offline performances (which are not accounted for in LADs), then the challenge is about where exactly is the student’s performance located? And who, in the event of failure, is responsible– the machine or the human?

In practice within, for example, a medical school context, we argue that this will likely be resolved in interaction, through configurations of the ‘microphysics of power’ (Foucault, 1977) in the performance of the relationship between the student, LAD and instructor. The LAD is deployed to stand in as the medical school’s mediator of judgement, which on occasion is supplemented by an instructor’s interpretation of it. The data displayed *is* the student. But what of the role of algorithm generating the connections between different forms of data that further construct the student’s performance? Is this a new ‘expert’ Rose (1999) within advanced liberal Technological Societies? Within the mainstream LAD literature this aspect of LADs seems to be ‘blackboxed’ (Akrich, 1992; Callon, 1990, 1992; Callon & Latour, 1982; Callon & Law, 1989), normalized to the point where it is just a natural part of the ecology of a health professions educational infrastructure that is transfer ready. This is an issue of:

‘…no matter how controversial their history, how complex their inner workings, how large the commercial or academic networks that hold them in place, only their input and output count’ (Latour, 1987, p.3).

In the case of the LAD in health professions education, input is interactivity (the diagram of social organization in technological societies (Barry, 2001) of the student, and output is the calculation of their learning performance. As an aside, maybe this kind of input and output is feasible with straightforward *events* (e.g., attended class) but we are challenged to see how complex, situated and interpretive learning *processes* such as interprofessional education and professionalism could be ‘flattened’ into quantifiable and comparable measures with any degree of fidelity.

**Fig. 4:**
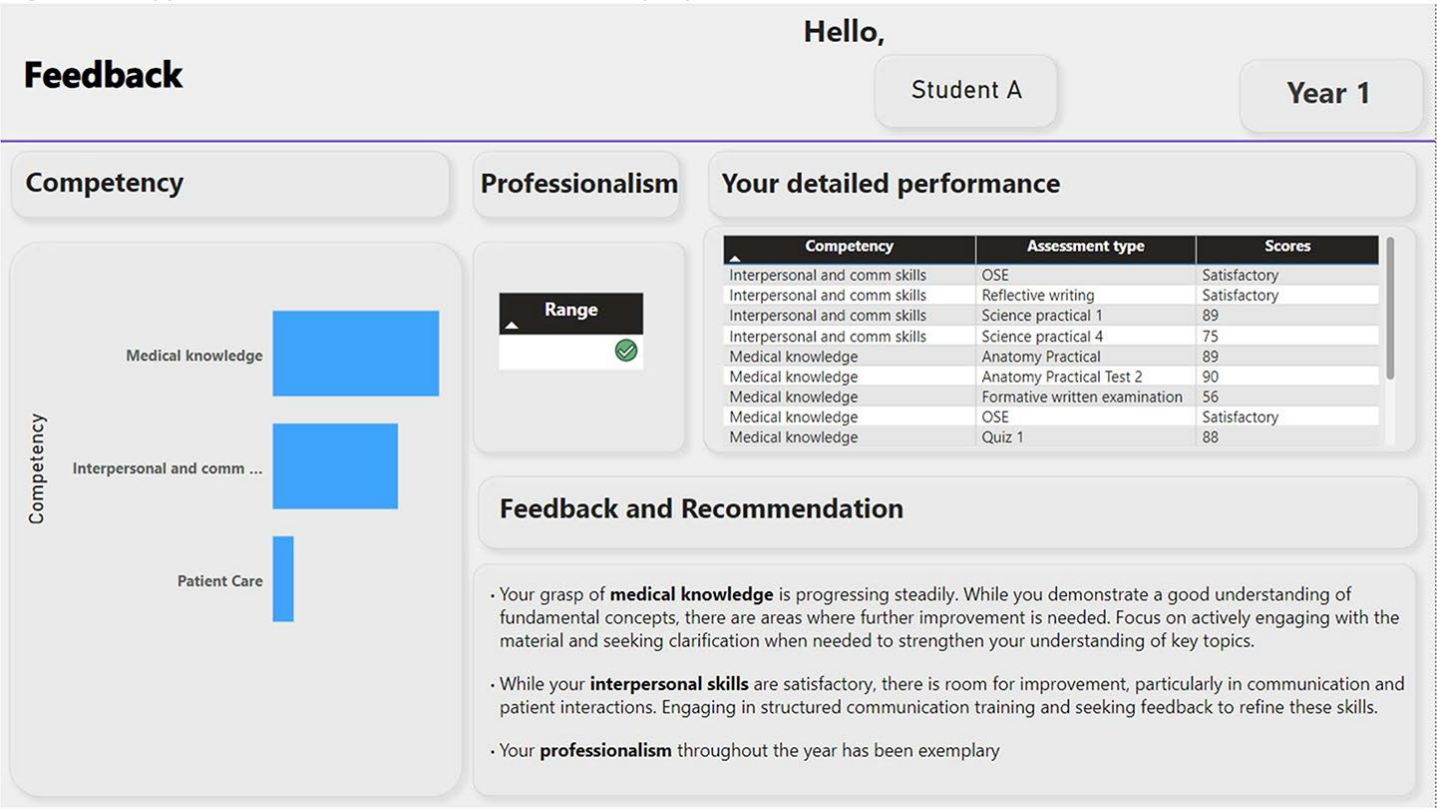
Hypothetical LAD Feedback Visual Display

In essence, in instances of the full automation of the feedback function in the LAD the role of ‘expert’ in advanced liberal Technological society is being delegated to the automated feedback functions, to non-human algorithms divorced of interpretation informed by context. The calculation of student performance and its representation in the visual displays coupled with recommendations is a ‘blackbox’ that the student, by themselves, cannot unpack (see example Fig. 4). The source and veracity of the learning recommendations cannot be questioned, cannot be negotiated with. In the context of a near future where Artificial Intelligence (AI) becomes central to the interpretation of data displayed on the LADs, the problematic situation of the unquestionable and unnegotiable recommendations of LADs could be compounded by the possibility of AI hallucinations relayed to student and instructor, in the form of questionable learning remediation recommendations.

From where we stand, full automation of the LAD feedback in medical education could produce three potential outcomes. Firstly, as outlined by Kitto et al. (2024) depending on the stage of education and training, learners may be more predisposed to LAD types of machine learning guidance (think transitioning into first clerkship years in UGME). Faced with the anxiety and uncertainty around the impossibility of mastery over constantly evolving healthcare knowledge and skills, uncertainty about their own gaps, knowledge, and uncertainties around variations in clinical instructor styles of practice and teaching, students looking for certainty in ways to progress through their degree may rush to uncritically embrace LAD guidance. To combat this tendency, inserting the socio-cultural context of health professions student learning could be a way to strengthen the connection between the LAD learning recommendations and compliance amongst students. The question is how to do this in a high-fidelity way, how can this be built into the LAD as a socio-technical assemblage? Given the ‘hallucination’ concern, teaching students how to account for the possibility of errors in the form of hallucinations and other shortcomings of nascent machine learning tools to avoid erroneous learning behaviour (Kitto, 2024) and in this case, avoid complying with mis-informed LAD learning recommendations, is critical. But in the case of the deployment of fully automated LAD learning recommendation algorithms (unmediated by humans), it is an impossibility as the source of guidance generated out of LADs is blackboxed, opaque and immutable.

Secondly, and conversely, there is the possibility of a breakdown in trust within student populations and the possibility of, “critical questioning [being] more likely to happen if the student has been given an underlying reason to be a little distrustful of the classifier” (Kitto et al., 2018, p.455). We suggest that this more than likely to occur within the learning analytics community which has yet to pursue the question of not only how classification schemas can shape worldviews and order human interactions in some ways, and simultaneously disorder them in others, but also in terms of how imperfect a classifier in and of themselves can be (see (Bowker & Star, 2000). In other words, in the case of lived reality of a student’s learning performance being mis-aligned with the calculations made by the LAD, their lived experience may trump LAD judgments and recommendations and lead to mistrust and non-compliant learning behaviours.

Thirdly, when data and analytics are regarded and lauded as neutral, objective and “as evidence of what will happen” (Prinsloo, 2017), students learn that there is only one version of reality when what data analytics provide is only one of many representations of reality. This is particularly problematic in health professions education given the weight that has already been allotted to evidence-based decision making in the field of medicine and healthcare. Outside the fields of medicine and healthcare, critics already recognise that transposing evidence-based management to education is limiting:

On the research side, evidence-based education seems to favor a technocratic model in which it is assumed that the only relevant research questions are questions about the effectiveness of educational means and techniques, forgetting, among other things, that what counts as ‘effective’ crucially depends on judgements about what is educationally desirable. On the practice side, evidence-based education seems to limit severely the opportunities for educational practitioners to make such judgements in a way that is sensitive to and relevant for their own contextualized settings (Biesta, 2007, p.5).

A health professions education facilitated by a technocratic approach to learning could exacerbate the positivist tendency inherent in evidence-based medicine and possibly reduce the student’s horizon of critical inquiry. Instead of relying on data and metrics to produce an ‘objective’ representation of reality (also referred to as a representationalist view), we could look to “a transactional view of evidence, data and analytics [which] acknowledges the incompleteness, the limitations and the possibility that the insights generated from analytics are, at best, provisional” (Prinsloo, 2019).

## Looking forward

So, drawing on our knowledge of the literature and the reflections presented to this point, what are we to make of this type of analytics-driven feedback? Whether automated or partially automated, LADs must be understood by all stakeholders as inherently imperfect and, as such, all stakeholders, especially students, should be empowered to engage productively with data-driven feedback rather than just being passive recipients (Kitto et al., 2018). The question then is: how are the conditions of this student ‘empowerment’ in relation to LADs to be constructed? Any contemplation of questions such as these must be addressed with the knowledge that, ‘technology both creates systems which close off other options and generates novel, unpredictable and indeed unthinkable, options (Callon, 1992).

These caveats should drive us toward doing more thoughtful empirical research in this topic area. Clearly, the situated performances of different LADs will produce different representations and content, different pedagogical and infrastructural conditions, and constraints. These nuances do matter: our point is that design, context and performance of a LAD will have predictable and unpredicable (un)intended effects. Therefore, there is a pressing need for empirical and critical analyses of the role and effects a LAD might have on learner performance and learner relationships with their peers, instructors or the educational institutions in which they are enrolled, and the implications of particular manifestations of a LAD thereon.

We have put forward one way to map out the educational effects through an explicit recognition of the LAD as situated within a Technological Society, a society that is information technology dense and highly political. When designing an LAD for health professions education, one must think beyond the technical and instrumental and consider one’s role in the designing of people, in this case healthcare professionals. These are now technological citizens, expected to be lifelong learners capable of operating in information technology dense healthcare systems, able to receive constant feedback and adapt. Grappling with the precariousness of the use of technical means to produce feedback systems that create an interactive type of citizen is, and will continue to be, a ‘wicked’ problem requiring constant reflection.

## Conclusion

In conclusion, we finish with a final argument that the core and ongoing question concerning feedback does not concern the creation of a ‘technical fix’ to the problem of more feedback (which students and educators often mistakenly cry out for), rather our attention should be focused on the nature, timing and deployment of *appropriate* feedback to the learner and their learning situation. Even more crucially, the creation of feedback content and systems must be designed in a way that is cognisant of their potential myriad unintended learning consequences, in order to be able to try and delimit them when and where possible. We suggest that looking through the lens of a Technological Society can help us this agenda whereby artefacts such as LADs can be more critically and deliberately engaged technically and politically in the construction of future healthcare professionals.

## Data Availability

No datasets were generated or analysed during the current study.
